# Arbovirus Detection in Insect Vectors by Rapid, High-Throughput Pyrosequencing

**DOI:** 10.1371/journal.pntd.0000878

**Published:** 2010-11-09

**Authors:** Kimberly A. Bishop-Lilly, Michael J. Turell, Kristin M. Willner, Amy Butani, Nichole M. E. Nolan, Shannon M. Lentz, Arya Akmal, Al Mateczun, Trupti N. Brahmbhatt, Shanmuga Sozhamannan, Chris A. Whitehouse, Timothy D. Read

**Affiliations:** 1 Biological Defense Research Directorate, Naval Medical Research Center, Silver Spring, Maryland, United States of America; 2 United States Army Medical Research Institute for Infectious Diseases, Fort Detrick, Maryland, United States of America; Duke University-National University of Singapore, Singapore

## Abstract

**Background:**

Despite the global threat caused by arthropod-borne viruses, there is not an efficient method for screening vector populations to detect novel viral sequences. Current viral detection and surveillance methods based on culture can be costly and time consuming and are predicated on prior knowledge of the etiologic agent, as they rely on specific oligonucleotide primers or antibodies. Therefore, these techniques may be unsuitable for situations when the causative agent of an outbreak is unknown.

**Methodology/Principal Findings:**

In this study we explored the use of high-throughput pyrosequencing for surveillance of arthropod-borne RNA viruses. Dengue virus, a member of the positive strand RNA *Flavivirus* family that is transmitted by several members of the *Aede*s genus of mosquitoes, was used as a model. *Aedes aegypti* mosquitoes experimentally infected with dengue virus type 1 (DENV-1) were pooled with noninfected mosquitoes to simulate samples derived from ongoing arbovirus surveillance programs. Using random-primed methods, total RNA was reverse-transcribed and resulting cDNA subjected to 454 pyrosequencing.

**Conclusions/Significance:**

In two types of samples, one with 5 adult mosquitoes infected with DENV-1- and the other with 1 DENV-1 infected mosquito and 4 noninfected mosquitoes, we identified DENV-1 DNA sequences. DENV-1 sequences were not detected in an uninfected control pool of 5 adult mosquitoes. We calculated the proportion of the *Ae. aegypti* metagenome contributed by each infecting Dengue virus genome (p^IP^), which ranged from 2.75×10^−8^ to 1.08×10^−7^. DENV-1 RNA was sufficiently concentrated in the mosquito that its detection was feasible using current high-throughput sequencing instrumentation. We also identified some of the components of the mosquito microflora on the basis of the sequence of expressed RNA. This included members of the bacterial genera *Pirellula* and *Asaia*, various fungi, and a potentially uncharacterized mycovirus.

## Author Summary

Traditional methods for virus detection often rely on specific attributes, such as DNA sequences, of the viruses and therefore they not only require *a priori* knowledge of the agent in question, but they also are generally very specific in nature, capable of detecting viruses only from within a specific family, for example. Nextgen sequencing shows much promise for detection/diagnostic applications because of its ever-increasing throughput, decreasing cost, and unbiased nature. We investigated the applicability of 454 pyrosequencing for viral surveillance of insect populations, using *Aedes aegypti* mosquitoes experimentally inoculated with Dengue virus type 1 (DENV-1) and calculated what proportion of the total nucleic acid from crushed mosquitoes was contributed by the virus. We concluded that 454 pyrosequencing is capable of detecting even very small amounts of a known virus from within a pool of infected and noninfected mosquitoes, but for the amount of sequencing reads required to detect the virus, this technique may currently be too cost-prohibitive for use in large-scale surveillance efforts. Interesting byproducts of our study included a glimpse into what symbiotic organisms *Ae. aegypti* may harbor, as well as what genes may be differentially expressed in a DENV-1-infected versus noninfected mosquito.

## Introduction

Dengue virus types 1–4 are emerging members of the genus *Flavivirus* in the *Flaviviridae* family, which consists of a group of related enveloped viruses with positive-stranded RNA genomes [1]. The four types can be distinguished through serologic assays, though each is capable of causing a spectrum of disease ranging from a mild or unapparent viral syndrome to severe and often deadly manifestations of hemorrhagic disease known as Dengue hemorrhagic fever (DHF) and Dengue shock syndrome (DSS). DHF is characterized by a sudden onset of fever with hemorrhagic complications such as petechiae and/or gastrointestinal hemorrhage and can be followed by shock and low blood pressure, hallmarks of DSS [2]. There is no approved specific therapeutic for dengue infection; treatment is limited to supportive care.

The viruses are transmitted throughout the tropics and subtropical areas by *Aedes aegypti*, a diurnal mosquito, and in South East Asia by a related species, *Ae. albopictus* [3], [4]. The estimated global burden of disease caused by dengue is 100 million cases per year with 250,000 to 300,000 cases of DHF annually, for which the case fatality rate is 5% [2]. Although dengue is arguably one of the more significant arthropod-borne (arbo-) viruses in terms of the morbidity and mortality it causes [5], it is not the only arbovirus that causes a significant threat to humans; West Nile virus, Japanese encephalitis virus, yellow fever virus, and others are also of major global concern [1]. Despite the ever-present global threat caused by arboviruses, there is not yet a single detection system that is capable of detecting all arboviruses simultaneously [6].

In general, traditional viral detection and surveillance methods can be costly and time consuming and generally require prior knowledge of the etiologic agent, as they rely on virus-specific primers or antibodies. Therefore, these techniques are unsuitable for situations when the causative agent of an outbreak is entirely novel or is an unknown sequence variant. The pitfalls of using specific PCR targets were vividly demonstrated when it was found that a new variant of a genital *Chlamydia trachomatis* strain had escaped detection for several years because it had acquired a deletion in the region of a virulence plasmid that was a target for a commonly used real-time PCR assay [7]. Recently, several groundbreaking studies have been published that used *de novo* high-throughput bead-based pyrosequencing of DNA [8] to provide putative identification of viral disease agents [9], [10], [11]. These studies all use a metagenomic approach [12]- sequencing a sample containing total nucleic acids- with no separation of host nucleic acids from those of the infecting or commensal microorganisms. In one such study, pyrosequencing was used to conduct a survey of microorganisms in honeybee colonies associated with colony collapse disorder (CCD) in comparison to colonies that were free of CCD [11]. In another study, organs were transplanted from the same donor to several recipient patients, all who died weeks later of a febrile illness. A variety of assays aimed at identifying the etiologic agent were uninformative. The authors of that study employed 454 pyrosequencing of the patients' samples and were able to identify, amidst a host background of 103,632 total reads, 14 reads corresponding to a novel, deadly arenavirus [9]. In a third recent study, a novel strain of Ebola virus was identified in Uganda by using pyrosequencing to examine patients' samples [10]. These three examples highlight the utility of pyrosequencing for relatively unbiased detection of an etiologic agent that is either unsuspected or novel, situations in which more traditional methods might not be appropriate. While this pioneering work highlights the promise of metagenomic sequencing based virus detection there has not been a study that measures the relationship of the number of DNA sequences detected to the number of virus particles present (although a recent study that utilized metagenomic sequencing of citrus phloem cells estimated the limit of detection for *Candidatus liberibacter asiaticus*, the bacterium thought to be the causative agent of Huanglongbing citrus disease, to be one bacterial cell per every 52 phloem cells [13]).

In the current study we explore the usefulness of pyrosequencing in the context of arboviral infections. *Ae. aegypti* mosquitoes experimentally infected with DENV-1 were pooled with noninfected mosquitoes to simulate samples that might be derived from real-world arbovirus surveillance programs. Using random-primed methods, total RNA was reverse-transcribed and the resulting cDNA subjected to high-throughput pyrosequencing. We successfully detected DENV-1 sequences from samples containing infected mosquitoes. Sequences matching DENV-1 were absent from a control sample of noninfected mosquito RNA. Based on the results from this survey we have produced a model of the type of sequencing capacity necessary to detect novel variants of dengue virus in screens of wild-caught *Ae. aegypti*.

## Methods

### Mosquito infections

*Aedes aegypti* (Rockefeller strain) were reared using standard methodology. Briefly, eggs were flooded, allowed to hatch overnight and resultant larvae were provided ground fish food and reared in a walk-in incubator maintained at 26°C. Pupae were removed and adults allowed to emerge into 3.8-liter cardboard cages with netting over the open end. Adult mosquitoes were transported to a Biological Safety Level-3 containment laboratory where they were inoculated intrathoracically with 0.3 uL of a DENV-1 (HAW strain) virus suspension containing 10^6.2^ PFU/mL of inoculum (10^2.7^ PFU/mosquito). Inoculated mosquitoes were maintained in an incubator at 26°C for 7 days. Mosquitoes were then killed by freezing at −20°C for 5 min and then triturated either individually or with the addition of uninfected *Ae. aegypti* in 0.6 mL of diluent (10% heat-inactivated fetal bovine serum in Medium 199 with Earle's salts [Invitrogen, Inc., Carlsbad, CA] and antibiotics). A 0.1-mL aliquot of the mosquito suspension was removed and added to 0.9 mL of diluent. This was frozen at −70°C until testing by plaque assay on Vero cells to determine the number of viral particles present in the sample. 1.5 mL of TRIzol-LS (Invitrogen, Inc., Carlsbad, CA) was then added to each tube and the contents of each vial were divided into two cryovials to create duplicate samples. TRIzol-LS extraction of total RNA from homogenized mosquitoes was performed according to the manufacturer's instructions. The final RNA pellet was resuspended in 50 uL of RNase-free water and stored at −70°C until use.

### PCR-ESI/MS

PCR-ESI/MS was performed as described previously. Briefly, RT-PCR was performed using an 8-primer pair pan-*Flavivirus* assay on the Ibis T-5000 [14]. The assay targets conserved genomic regions of the known pathogenic mosquito- and tick-borne members of the genus *Flavivirus*. After PCR, the reactions were desalted, and the purified DNA products were individually sprayed into a Bruker Daltonics microToF (Billerica, MA) mass spectrometer. Proprietary signal-processing software was used to deconvolute raw data from the mass per charge. This molecular mass was then assigned to the amplicon's empirical molecular mass and correlating base composition, which was matched with those in the system's database. For every RT-PCR reaction well, the signal amplitude of the internal positive control (which is spiked into every reaction at a known concentration of 100 copies per reaction) and the sample were compared and interpreted to give quantitative results.

Mosquito suspensions were tested for infectious virus by plaque assay on MK-2 (monkey kidney) cells. Briefly, serial 10-fold dilutions of the mosquito suspension were made in diluent and 0.1 mL added to each well of a 6-well plastic tissue culture plate. An agarose overlay was added 1 hour later and a second overlay, containing neutral red, added 5 days later. Plaques were enumerated the following day.

### Metagenomic sequencing

Methods in a 454 Application Note for sequencing Influenza Virus RNA [15] were modified slightly to be used for detecting flavivirus RNA amidst insect RNA at concentrations relevant to experimental inoculation. The most substantial modification to the procedure was the starting material; rather than starting from purified viral nucleic acid, total RNA (that of mosquito, viral, and potential commensal organisms) was used as input material. Enrichment for viral RNA via a biotin-labeled oligonucleotide specific for viral sequences was not performed. Additionally, other small differences in our procedure as compared to that of the Application Note consisted of using random heptamer primers for reverse transcription, other slightly modified oligo sequences, and omission of the second round of RNA Clean and AMPure (Agencourt Biosciences Corporation, Beverly, MA) bead purifications. Briefly, total mosquito RNA was fragmented at 82°C for 2 minutes in fragmentation buffer as described in the Application Note, purified with one round of RNAClean, and then RT-PCR was performed using a random heptamer primer (5′-phosphate-N_7_-3′). RT-PCR conditions, RNA removal, reaction neutralization, and single-stranded cDNA recovery were performed as per the Application Note.

In preparation for FLX sequencing, adaptors with overhangs were made by annealing together pairs of complimentary oligos. Adaptor A was made by annealing the following 2 oligos together: mod sscDNA OligoA′ 5′-NNN NNN CTG ATG GCG CGA GGG AGG-dideoxyC-3′ and sscDNA OligoA 5′-GCC TCC CTC GCG CCA TCA G-3′, and Adaptor B was made by annealing together the following 2 oligos: sscDNA OligoB 5′-biotin-GCC TTG CCA GCC CGC TCA GNN NNN N-phosphate-3′ and sscDNA OligoB′ 5′-phosphate-CTG AGC GGG CTG GCA AGG-dideoxyC-3′. Annealing and directional ligation of the adaptors onto cDNA was performed essentially as described in the Application Note. The final cDNA library was subjected to only one round of RNAClean.

An amplification PCR was performed prior to emulsion PCR using the following oligos: Amplification Primer A 5′-GCC TCC CTC GCG CCA-3′ and Amplification Primer B 5′ GCC TTG CCA GCC CGC-3′, Advantage 2 polymerase mix (Clontech, Mountain View, CA), and the PCR conditions described in the Application Note. All oligos were purchased from Invitrogen (Carlsbad, CA). Emulsion PCR was performed using 454 emPCR kits II and III (Roche, Indianapolis, IN). After enrichment, the resulting emPCR II- and III-derived beads were pooled 1∶1 for the sequencing run.

### Ribosomal RNA depletion

A 9 ul (∼600 ng) aliquot of the sample in question (consisting of total RNA extracted from a pool of 5 male *Ae. aegypti* experimentally inoculated with DENV-1) was taken and subjected to eukaryotic ribosomal RNA depletion using the RiboMinus Kit (Invitrogen) followed by ethanol precipitation with glycogen (Applied Biosystems/Ambion, Austin, TX) as a carrier for lower molecular weight RNA species. After the ethanol-precipitated, rRNA-depleted, RNA was air-dried and resuspended in DEPC-treated water, it was fragmented, reverse-transcribed and sequenced as described in the metagenomic sequencing protocol (above). For sake of comparison, a second aliquot (3 ul) of the same total RNA prep was also fragmented, reverse-transcribed, sequenced, and analyzed in parallel, with the exception that this second aliquot was not subjected to rRNA depletion. For the rRNA depletion experiments, BLASTn was used to find hits to *Ae. aegypti* and *Ae. albopictus* rRNA genes as well as DENV-1 genome (GenBank Accession DVU88536; BLAST cutoff used was bit score greater than 100).

### Reference and transcript mapping

Transcript analysis and remapping analyses were performed using CLC Genomics Workbench v3.7 (CLC Inc, Aarhus, Denmark). The *Ae. aegypti* whole genome shotgun data [Genbank: AEGE00000000] was downloaded in December 2009. CLC Reference assembler was run with Insertion cost = 3, Deletion cost = 2 and Mismatch cost = 2. Contigs with BLAST match scores greater than 100 to the *Ae. albopictus* rRNA operon [Genbank: MQSRAGN] were flagged as probably containing *Ae. aegypti* rRNA operons. The AaegL1.2 collection of 18,760 known transcripts was downloaded from VectorBase (www.vectorbase.org). CLC RNA-seq was run with Minimum length fraction of 0.9 and Minimum exon coverage fraction of 0.2. GS Reference Mapper software (v2.0.01.14; Roche/454) was used to produce reference-guided assemblies of each of the mosquito datasets with respect to the DENV-1 genome (GenBank; DVU88536) and those results were viewed in Geneious software (Biomatters Ltd., Auckland, New Zealand).

### Analysis of the mosquito microflora

Contigs and individual reads were compared to sequences in NCBI nucleotide (nt), refseq_RNA, environmental sequences (env_nt), and whole genome sequence (WGS) databases using megaBLAST and protein (nr) database using BLASTx. The best BLAST hit was chosen and reported based on e value and sequence identity. Individual reads for each library were also uploaded to the MG-RAST server at http://metagenomics.nmpdr.org and are publicly accessible, listed under the identifiers 4441794.3 (N2187), 4441733.3 (N2173), 4441793.3 (N2175), 4450608.3 (N2473), and 4450615.3 (N2474). For the bacterial sequences identified, phylogenetic profiles were examined and most numerous hits were reported based on the RDP or SEED datasets. Minimum alignment length used was 50 and maximum e-value was set at 0.01.

## Results

### 454 shotgun sequencing of Dengue-infected mosquitoes

In order to assess 454 pyrosequencing as a platform for detection of arboviruses in their insect vectors, adult female *Ae. aegypti* were inoculated with 0.3 uL of a suspension containing 10^6.2^ plaque-forming units (PFU)/mL (a total of 10^2.7^ PFU per mosquito). These mosquitoes were held at 27°C for 7–12 days and then mixed with noninfected control mosquitoes to simulate samples that might result from real-world arbovirus surveillance programs. The pooled mosquitoes were triturated and total RNA was extracted using TRIzol and reverse-transcribed using random primers. 454 GS FLX libraries were constructed from the resulting total cDNA (see materials and methods). Library N2173 was made from five DENV-1-infected mosquitoes, library N2187 was made from 5 noninfected control mosquitoes, and library N2175 was made from five pooled mosquitoes, only one of which was infected with DENV-1. These three FLX libraries were sequenced individually using the 454 GS FLX sequencer and each run resulted in roughly 200,000 to 350,000 reads per library post 454 quality filtering- roughly 30–60% less reads than would be expected from a whole genome sequencing run on the FLX instrument. The average read length was 208 bases long, noticeably shorter than the average read length generally produced from GS FLX sequencing of genomic DNA (∼250 bases), likely a result of the heat fragmentation step used to create appropriate sized RNA fragments, in place of the usual nebulization step involved in sequencing genomic DNA. In the case of these mosquito cDNA libraries, the average size of DNA fragments before binding adaptors was 150 bases, while for standard FLX libraries made from DNA fragmented via nebulization with Nitrogen gas, the average DNA fragment size is generally around 200 bases. Post sequencing, the resulting reads were assembled *de novo* into contigs using the 454 Newbler assembler with default parameters (minimum overlap length of 40 and minimum overlap identity of 90). A relatively small number (less than 4%) of the overall reads assembled into large contigs (those greater than 500 nucleotides in length).

### Detection of DENV-1

**Figure 1:**
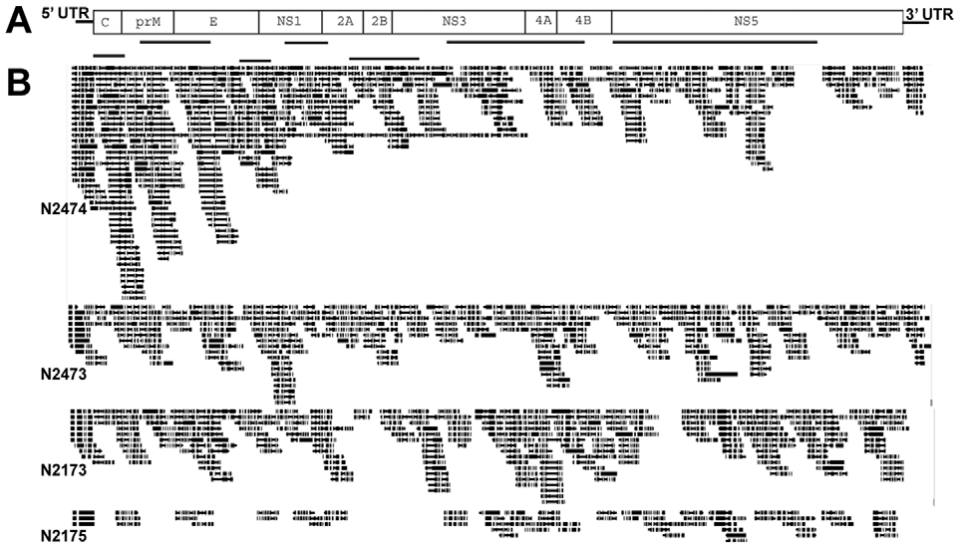
Coverage of Dengue genome. A) Reads resulting from sequencing library N2173 were assembled *de novo* using the Newbler Assembler (454/Roche) and then the 23 large contigs (greater than 500 nucleotides) were subjected to BLAST analysis to determine their best hits. The 7 Dengue-specific large contigs are shown here as horizontal lines below regions of the Dengue genome they correspond to. Scale is approximate. B) Reads resulting from sequencing of all 5 libraries were mapped to the DENV-1 genome using GS Reference Mapper (454/Roche) and then viewed using Geneious software. The control noninfected mosquito dataset had no reads that mapped to the DENV-1 genome and therefore is not shown here.

As no physical means were used to purify or separate by origin the various RNA species present in each sample, it was expected that mosquito ribosomal RNA would make up a large percentage of the total. In fact, upon analysis of the resulting reads, *Ae. aegypti* ribosomal RNA was indeed found to constitute a significant percentage of the resulting reads, making up as much as 92% of the reads in the case of the N2173 library (Table 1). (For a detailed analysis of which de novo assembled contigs from each dataset map to mosquito ribosomal RNA, see Tables S5, S6, S7, S8.) However, despite the large contribution of rRNA, DENV-1-specific reads were still detected. The individual reads from each library were aligned against the NCBI nonredundant nucleotide and protein databases using BLASTn or BLASTx respectively [16] and additionally, reads from each of the 3 datasets were also assembled using the DENV-1 genome as a reference and results of the latter are displayed in Figure 1B. In the case of the library constructed from 5 infected mosquitoes (N2173), there were 227 reads (0.08% of the total number of reads) that mapped to the DENV-1 genome (Table 2). Consistent with the relative numbers of infected mosquitoes there were proportionately 5 times fewer DENV-1 hits in the dataset from the library made from 1 infected mosquito pooled with 4 noninfected mosquitoes (N2175), indicating that the relative percentage of virus-specific hits per sequencing run directly correlates with the proportion of infected to noninfected mosquitoes and titer of virus present in a sample of pooled insects. The DENV-1 matches for these two datasets were relatively well spaced over the entire Dengue genome, suggesting that there was no significant inherent bias toward one end of the genome over the other in efficiency of reverse-transcription or adaptor ligation (Figure 1).

**Table 1:** Major constituents of *Ae. aegypti* metagenome are listed below, with number of reads corresponding to each category as well as percentage of the total reads.

	5 infected, F		1/5 infected, F		5 noninfected, F		5 infected, M		5 infected, M	
	N2173		N2175		N2187		N2473		N2474	
	reads	% reads	reads	% reads	reads	% reads	reads	% reads	reads	% reads
***Ae. aegypti*** ** rRNA**	268,015	92.60	182,788	84.56	246,080	72.03	239,991	61.38	183,687	54.54
***Ae. aegypti*** ** transcripts**	2,353	0.81	2,268	1.05	15,559	4.55	1,915	0.49	4,574	1.36
**Bacterial**	8,567	2.96	12,663	5.86	14,656	4.29	39,615	10.13	22,119	6.57
**Fungal**	1,559	0.54	2,988	1.38	2,041	0.60	8,897	2.28	2,862	0.85
**Other**	8,942	3.09	15,457	7.15	63,314	18.53	100,553	25.72	123,580	36.69
**Total reads**	289,436		216,164		341,650		390,971		336,822	

Number of reads corresponding to *Ae. aegypti* rRNA and *Ae. aegypti* transcripts were determined by BLAST and CLC workbench. Number of reads derived from bacteria and fungi were determined using MR-RAST RDP and MG-RAST SEED analysis, respectively.

**Table 2:** Results of reference-guided assembly.

	N2473	N2474	N2173	N2175
	5 infected, M		5 infected, F	1 of 5 infected, F
rRNA depletion	−	+	−	−
**number reads mapped**	243	419	227	46
**number bases in assembly**	10,412	10,106	9,699	6,282
**% DENV-1 genome covered**	97.0%	94.1%	90.3%	58.5%
**number large contigs**	7	3	7	3
**total contigs**	7	5	8	15
**N50 contig size (nt)**	1,635	3,197	2,050	758
**largest contig size (nt)**	2,272	5,590	2,366	1,149
**average depth of coverage**	4.5	8.3	4.9	1

Each of the 5 datasets were assembled with GS Reference Mapper software using the DENV-1 genome as a reference. Statistics obtained for each of the 4 datasets containing DENV-1 specific reads are shown here. The N2187 control noninfected mosquito dataset was also mapped to the DENV-1 genome, but resulted in no assembled reads.

The large contigs produced by *de novo* assembly of the libraries were queried against the NCBI nucleotide (nt), refseq_RNA, environmental sequences (env_nt), and whole genome sequence (WGS) databases using megaBLAST or the protein (nr) database using BLASTx. For N2173, the library made from 5 infected mosquitoes, 7 of 23 large contigs were found to be DENV-1-specific (Figure 1A). The total sequence encompassed by those 7 large contigs was 8517 bases (212 reads), representing 79.3% coverage of the 10.7 kB viral genome in just the large contigs alone. By comparison, for N2175, the library created from 1 infected out of 5 total mosquitoes, only 2 of the 23 large contigs (made up of 21 reads) were DENV-1-specific, and for N2187, the library made from 5 noninfected mosquitoes, none of the 38 large contigs were a match to the DENV-1 genome.

Not surprisingly, reference-guided assembly of the reads resulted in contigs with greater coverage of the Dengue genome and included more DENV-1 reads than were included in the *de novo* assembled large contigs. Average depth of coverage of the DENV-1 genome scaled with the proportion of mosquitoes infected with DENV-1, being 4.9× for N2173 (5 infected) and 1× for N2175 (1 of 5 infected; Table 2).

### rRNA depletion

As such a large proportion of each dataset was found to consist of (mainly host) ribosomal RNA sequence, we sought to decrease the proportion of eukaryotic ribosomal RNA sequences and thereby improve the sensitivity of detection of DENV-1. For this experiment, starting material consisted of total RNA extracted from a pool of 5 male *Ae. aegypti* experimentally inoculated with DENV-1. An aliquot of this RNA sample was treated using a commercially available kit that consists of biotinylated bead-bound probes designed to hybridize and selectively deplete eukaryotic rRNA (mouse and human). Following this treatment, the rRNA-depleted sample (N2473) was reverse-transcribed and sequenced as described in the methods. In parallel, a second aliquot of this same RNA sample (N2474) was also reverse-transcribed and sequenced, but without being subjected to rRNA depletion.

The sequence quality and output of each of the two sequencing runs was overall similar, producing 391,103 and 336,905 reads respectively. As expected, in the N2474 dataset, there was an overall decrease in the total number and proportion of reads corresponding to Aedes rRNA sequences (proportion decreased from 61.4% to 54.5%) and an increase in the depth of coverage of the DENV-1 genome (Table 2). Each of the five datasets were aligned to the DENV-1 genome and results are shown in Figure 1B. This reference-guided assembly of the N2473 dataset demonstrates 4.5× average depth of coverage of the DENV-1 genome as compared to 8.3× average depth of coverage by the N2474 dataset. Notably, although rRNA depletion resulted in a higher average depth of coverage, the coverage was skewed toward the 5′ end of the DENV-1 genome.

### Estimation of the proportion of reads contributed by the infecting Dengue viruses

From the sequencing data above, we estimated the proportion of reads attributable to a single infecting DENV-1 pathogen in each experiment, a number we called “p^IP^”. This was done by dividing the proportion of DENV-1 reads found in each experiment by an estimate of the number of infecting viruses (Table S9). The number of DENV-1 genomes per mosquito was assessed both by testing an aliquot of each sample using PCR electrospray ionization mass spectrometry (PCR-ESI/MS) and the Ibis T5000 biosensor or using titration on MK2 cells. Based on the IBIS data the p^IP^ ranged from as low as 2.75×10^−8^ for N2173 to 1.08×10^−7^ for the depleted N2474 experiment. The empirically derived p^IP^ value can be useful for planning future experiments that screen mosquitoes for DENV-1 like viruses by shotgun sequencing (see discussion).

### RNA expression profile of Dengue-infected and noninfected mosquitoes

For comparison the 100 most abundantly expressed transcripts in each female mosquito sample were ranked by RPKM value (the number of reads which map per kilobase of exon model per million mapped reads, as in [17]; see Tables S1, S2, S3, S4). Overall, each sample shared about 50% similarity in the top ranked expression list. Six highly expressed transcripts that were in the top ten for all three libraries (Table 3) were AAEL017647-RA and AAEL017654-RA, both of which are ribosomal RNA; AAEL017811-RA, RNase MRP; AAEL017868-RA, eukaryotic type signal recognition particle; and AAEL001702-RA and AAEL017571-RA both of which have no description in VectorBase. Relatively few genes known to be associated with a function were differentially expressed in the N2173 sample versus the other samples. An exception was a putative salivary gland mucin [GenBank: DQ440007.1] [18], which was found to be up-regulated and may represent a host defense response to Dengue virus infection. Other exceptions include down-regulated transcripts corresponding to trypsin 3a1 and up-regulated transcripts corresponding to tRNA^Gly^. The Dengue-infected mosquito RNA contained a higher percentage of *Ae. aegypti* rRNA transcripts and correspondingly lower percentage of transcripts from other genes. Overproduction of rRNA in response to viral infection has been previously reported in silk worms infected with cytoplasmic polyhedrosis virus [19] and human liver-derived cells infected with Hepatitis C virus [20] and may represent a physiological response to viral stress on the mosquito cells or may be the result of viral takeover of the host protein making machinery, although the true biological implications of this finding are not clear at this time. The top expressed transcript in both the infected samples was AAEL017413-RA, a transcript for which there is no description in VectorBase, but upon BLAST analysis, bears significant similarity to large subunit ribosomal RNA.

**Table 3:** Selected transcript profiles in DENV-1 infected and noninfected mosquitoes.

		5 noninfected			1 of 5 infected			5 infected		
		N2187			N2175			N2173		
transcript	Vectorbase putative product	# reads	RPKM	rank	# reads	RPKM	rank	# reads	RPKM	rank
AAEL017413-RA	no description	14	2,243.73	24	125	137,786.60	1	643	683,170.42	1
AAEL017647-RA	5.8S ribosomal RNA	29	12,150.94	4	8	23,054.49	4	18	49,998.75	2
AAEL017811-RA	RNase MRP	227	48,669.62	2	27	39,815.26	2	29	41,219.71	3
AAEL017868-RA	eukaryotic type signal	190	41,289.04	3	13	19,430.25	5	17	24,490.91	4
	recognition particle RNA									
AAEL017654-RA	small subunit ribosomal RNA,	42	4,833.90	10	21	16,623.45	6	21	16,022.94	5
	5′ domain									
AAEL001702-RA	conserved hypothetical protein	95	11,871.61	5	15	12,892.31	7	15	12,426.59	6
AAEL017571-RA	no description	3617	122,231.86	1	109	25,334.72	3	35	7,841.13	7
AAEL017247-RA	no description	10	3,391.89	13	1	2,332.89	30	3	6,745.86	8
AAEL016633-RA	tRNA^Gly^	0	NA	NA	0	NA	NA	1	5,902.63	9
AAEL016690-RA	tRNA^Gly^	0	NA	NA	0	NA	NA	1	5,902.63	10
AAEL017898-RA	nuclear RNAse P	38	6,619.71	8	2	1,198.14	88	1	1,154.86	71
AAEL007818-RB	no description	38	9,957.61	6	4	1,999.62	41	2	963.69	91
AAEL007818-RA	trypsin 3A1	38	2,777.71	17	2	1,005.51	NA	2	969.19	89
AAEL017805-RA	U5 splicesomal RNA	18	9,015.00	7	0	NA	NA	0	NA	NA
AAEL017646-RA	U1 splicesomal RNA	14	5,472.52	9	1	2,688.52	24	1	2,591.40	26
AAEL017841-RA	U1 splicesomal RNA	0	NA	NA	0	NA	NA	2	5,182.80	12

Selected *Ae. aegypti* transcripts that were detected are listed along with their putative gene functions as reported in VectorBase, the number of total reads that correspond to each transcript, RPKM value (a normalized measure of expression level reported by CLC Workbench and which represents the number of reads that map per kilobase of exon model per million mapped reads), and the rank order of each transcript per library.

### Identification of symbiotic microorganisms

The shotgun sequencing of cDNA derived from triturated insect total RNA is in essence a metagenomic experiment, and so it was expected that the results of this sequencing could include identification of various bacterial or other types of symbiotic microorganisms living on or in the mosquitoes. In order to more fully characterize the diversity of organisms present in these mosquito samples, the datasets were analyzed using MG-RAST, a publicly available web service with a pipeline that analyzes user-uploaded metagenomic datasets and provides automated metabolic and phylogenetic reconstructions according to various parameters that can be adjusted by the user [21]. The reads were compared against the RDP dataset of ribosomal RNA (rRNA) sequences [22] and thresholds were set for a minimum alignment length of 50 and maximum E-value of 0.01. Based on this automated phylogenetic analysis of the reads, bacterial ribosomal RNA was found to constitute roughly 3 to 6.5% of the total number of classifiable reads for each library's dataset (without treatment to reduce the contribution of eukaryotic rRNA). Specific bacterial taxa with the highest number of hits were the two genera *Pirellula* and *Asaia*. Additionally, unclassified bacteria, including unclassified Clostridiales and unclassified *Enterobacteriaceae*, were found to constitute a relatively large percentage of the total bacterial rRNA contribution (Results are summarized in Table S10).

Based on the alignment of reads against the RDP database, as well as BLAST analysis of the large contigs, as much as 0.12% of reads from a given mosquito pool had their best hit to rRNA from members of the genus *Asaia*. This may likely indicate that all of the *Ae. aegypti* mosquitoes used in this study were infected or colonized with *Asaia* sp., regardless of their DENV-1-infected status. However, as there is currently no fully sequenced genome from a member of the genus *Asaia* available in GenBank for comparison, it is conceivable that the total number of *Asaia*-specific reads in our dataset could be underestimated.

In addition to the various bacterial rRNA sequences, fungal rRNA was found to constitute between 0.6% and 2.3% of the total reads in each library's dataset (using the SEED algorithm with a minimal alignment length of 50 and maximum e-value of 0.01). With these parameters, the top hit was to *Saccharomyces cerevisiae*. *De novo* assembly resulted in large contigs that by BLAST had their best hit to a rRNA of various fungal origins (including the genera *Penicillium* and *Aspergillus*) and in all five samples an RNA-dependent RNA polymerase encoded by a mycovirus was identified by SEED analysis of reads using MG-RAST and in 2/3 cases examined, by BLAST analysis of large contigs as well.

## Discussion

In this study we examined the feasibility of detecting a known RNA arbovirus directly from its host or vector via an unbiased and high-throughput method that obviates the need for culture methods or for prior knowledge of the etiologic agent. For this purpose, we utilized random-primed reverse transcription followed by 454 pyrosequencing to amplify and sequence total RNA extracted from pools of mosquitoes, some of which were experimentally inoculated with DENV-1. Although as expected, mosquito ribosomal RNA made up the vast majority of sequences present in the samples, using straightforward bioinformatics methods we were able to detect DENV-1-derived sequences present at relatively low levels. In fact, DENV-1 was even detected in the sample that consisted of only 1 infected mosquito and 4 noninfected. The total number of DENV-1-specific reads from each sample and the proportion of DENV-1-specific reads in relation to other reads derived from each sample were both found to be directly proportional to the number of DENV-1-infected mosquitoes and titer of virus in each sample, indicating that our random-primed RT-PCR was able to amplify all RNA species in an efficient and unbiased manner, including even those that were present at extremely low levels (0.1%) as compared to the rest of the sample. In an attempt to decrease the contribution of host rRNA to the read pool, we used a commercially available kit to selectively deplete eukaryotic rRNA and found that the average depth of coverage of the DENV-1 genome increased considerably.

Data we have presented here, where DENV-1-derived reads constitute much less than 0.1% of the total metagenome, and other studies, where Arenavirus reads constitute 0.01% of the reads derived from sequencing of a human clinical sample [9], show that infecting viral reads make up a small portion of the total metagenome. This assumption is also further supported by other metagenomic sequencing experiments performed in our laboratory where we have been able to detect viruses, whether novel or otherwise, by sequencing metagenomes from various species without physical enrichment of the nucleic acid for viral as opposed to host material, but in all cases, the virus-derived reads have constituted only a very small fraction of the total material (unpublished data). How can these data be used to eventually design experiments on wild-caught insects? One approach is to assume that each sequence read is a random sampling of the total metagenome that can be modeled by the Poisson distribution (1).
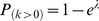
(1)Where 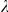 is the mean number of events and P_(k>0)_ is the probably of at least one event (finding a DENV-1-derived read). The equation can be re-arranged as,
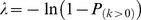
(2)An event, in this case, is the finding of at least one DENV-1-derived read amongst all the reads in the sequencing project. In this example, it is assumed that the sample contains only one infected insect. Therefore 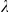 can be computed as the product of the total number of reads obtained (N_r_), the empirically derived number for the proportion of the reads attributable to one infecting virus genome (p^IP^) and the presumed number of pathogen genomes infecting one host (N_p_), divided by the number of hosts in the study (N_h_).
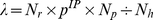
(3)Combining (2) and (3),
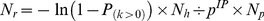
(4)
Table 4 outlines the hypothetical results of a Dengue screening experiment, involving an N_h_ of 100 wild-caught mosquitoes, where the investigator is looking to detect a minimum of one insect being infected. The N_p_ number, the titer of Dengue virus for a naturally infected mosquito, has to determined empirically but is thought to range from 10^2^ for a midgut infection to 10^5^ for a disseminated infection (M. Turell, unpublished data). Using a p^IP^ value estimated in this study, the number of sequences required for detection ranges from the billions to detect very low level infection on one hand to approximately 20,000 for 95% chance of detecting a mosquito with disseminated infection in a rRNA depleted sample. It is important to remember that at this stage, the p^IP^ values determined in these experiments are rough first estimates of this figure, and will be improved by subsequent sampling.

**Table 4:** Sequence read depth required for hypothetical sequencing based Dengue virus-like screening experiments.

N_p_	p^IP^ = 1.08E-7	p^IP^ = 1.08E-7	p^IP^ = 2.75E-8	p^IP^ = 2.75E-8
	P = 0.99	P = 0.95	P = 0.99	P = 0.95
10	4.26E+08	2.77E+08	1.67E+09	1.09E+09
50	8.53E+07	5.55E+07	3.35E+08	2.18E+08
100	4.26E+07	2.77E+07	1.67E+08	1.09E+08
1,000	4.26E06	2.77E+06	1.67E+07	1.09E+07
10,000	4.26E+05	2.77E+05	1.67E+06	1.09E+06

Values in the table are number of reads required (under the assumption that only one read is sufficient to identify the virus) to detect one infected mosquito in 100 (N_h_), under different assumptions of the number of infecting viruses (N_p_). Four different scenarios are presented, based on the high and low values on p^IP^ calculated in this study (N2474, the rRNA depleted sample and N2173) and power to detect of 95% and 99%. Values were calculated using equation 4.

At the present time all next generation sequencing technologies are too expensive and slow for use in routine screening. 454 pyrosequencing cannot economically be used in experiments to generate tens of millions of reads and the ‘short read’ (50–200 nt) platforms such as the ABI SOLiD 4 s or Illumina Hi-Seq have a maximum effective yield in the billions of reads. Depletion of host RNA, as shown by these studies, can bring down the effective number of reads needed for detection. The cost versus benefit of the additional time and expense involved in running depletion studies needs to be viewed in terms of the estimated depth of coverage necessary to achieve experimental aims (Table 4).

The model presented above assumes that all virus reads would be detected by significant matches against a reference database, and this may not necessarily be the case if sequencing technologies that produce short reads (especially <50 nt [23] are deployed for detection and the virus sequence is significantly different as compared to others in the database. It will be especially difficult to detect novel arboviruses that have a nucleic acid sequence very different from any previously characterized virus. Our ability to do so could in part hinge on whether or not or how well the reads assemble into contigs that can be used for BLASTx analysis. Experiments aimed at determining how well high-throughput pyrosequencing will perform in detection of novel arboviruses in their insect vectors are currently under way in our laboratory.

A second aim of this study was to obtain a metagenomic profile of Dengue-infected and noninfected *Ae. aegypti*. Based on rRNA analysis, the largest contribution of bacterial rRNA in all 3 of the mosquito samples, regardless of their virus-infected status, was that of unclassified bacteria. This is a rather interesting result, although the significance of this finding is not clear at this time. On the other hand, the two specific bacterial genera found to be most abundant were *Pirellul*a and *Asaia*. While bacteria in the genus *Asaia* have been previously reported to colonize *Anopheles* sp. mosquitoes [24], to our knowledge, they have not previously been reported as living in association with Aedes mosquitoes. The finding that reads derived from the α-proteobacterial genus *Asaia* constitute such a significant proportion of the overall reads is particularly interesting in light of what is know regarding the relationship of *Asaia* sp. with other types of mosquitoes. *Asaia* sp. are a group of acetic acid bacteria that, although reported as a stable colonizer of wild-caught and laboratory-bred Anopheles mosquitoes [24], have never before been reported as living in association with *Ae. aegypti*. Like *Wolbachia spp*, bacteria that live as endosymbionts with *Culex* mosquitoes and other insects [25], they have been proposed for use as an agent to control *Anopheles* [26]. *Asaia* sp. have also been proposed for use as a malarial control agent due to their ability to efficiently cross mosquito body barriers and persist within the insect [24]. The detection of a relevant mosquito symbiont as the contributor of a significant proportion of the total reads resulting from these three sequencing libraries strongly suggests that the bacterial rRNA sequences reported here truly are representative of the mosquito metagenome, and were not accidentally introduced as nucleic acid contaminants. Additionally, the findings of this study suggest that *Asaia* sp. could not only be investigated for use as a potential agent for controlling the spread of malaria by *Anopheles* mosquitoes, but could also be investigated for use in control of dengue transmission by *Aedes* mosquitoes as well.

To our knowledge, this is the first report of *Pirellula* sp. being found in association with mosquitoes. *Pirellula* is a genus of bacteria within the order Planctomycetales, a unique grouping of bacteria that have an unusual morphology, relatively controversial origin [27], [28], and about which relatively little is known due to the difficulty involved in culturing them and the subsequent paucity of strains that have been isolated [29]. Pirellula have been previously isolated from the giant tiger prawn [30] and subsequently the genomes of several *Planctomycetes* sequenced [31], [32].

Another result of this study is a glimpse into the transcriptome of DENV-1 infected versus noninfected *Ae. aegypti* mosquitoes. Although relatively few conclusions can be drawn due to the small sample number, several interesting observations were made. Firstly, levels of host ribosomal RNA were found to be increased in infected mosquitoes as compared to their noninfected counterparts. It is possible that this is due to a virally driven increase in protein synthesis. Another set of transcripts found to be up-regulated in the virus-infected samples was that which code for tRNA^Gly^. It has been shown that some viruses, such as Human Immunodeficiency Virus (HIV) can utilize tRNA_3_^Lys^ as a primer for transcription [33]–[34], but whether or how tRNAs play a special role in the lifecycle of dengue viruses is unclear. The finding of a possible down-regulation of a gene encoding a salivary gland mucin is notable in the light of a previous study by Girard et al in which expression of a salivary gland mucin of *Culex quinquefasciatus* was also found to be modulated in response to Flavivirus infection, although in that particular case, the mosquito/virus model is different (*Cx. quinquefasciatus*/West Nile virus) and the mucin expression was decreased [35]. The disparate findings between that work and the present study may serve to highlight the importance of salivary gland mucins overall to the outcome of an arbovirus infection and the very closely tailored relationship between a particular virus and its vector. Yet another intriguing observation was that trypsin 3A1 appears to be down-regulated by roughly 3 fold in DENV-1 infected mosquitoes. This may represent a notable finding as other researchers have reported that midgut trypsins condition *Ae. aegypti*'s body for DENV-2 infection [36], addition of exogenous trypsin or pretreatment of DENV-2 with trypsin slows dengue replication in *Ae. aegypti* [37] and that a gene encoding a trypsin inhibitor is expressed differentially in DENV-2-refractory mosquitoes relative to DENV-2-sensitive mosquitoes [38].

In conclusion, results presented here represent a proof-of-principle experiment—an initial step toward developing a real capability to ask complex questions regarding pathogen-vector dynamics and performing unbiased multi-agent surveillance directly within a vector or host itself. Taken in conjunction with the results of several other recent studies using similar methods [9], [10], [11], these data suggest that sequencing of new viruses may in the future be performed directly from initial samples and need not be dependent on the time-consuming work involved in culturing a new virus. If this type of sequencing were eventually integrated into the clinical laboratory, the potential savings in time to diagnosis in mysterious or unusual cases could have beneficial effects in terms of faster treatment or response time. Potential uses for this type of sequencing workflow are not limited to RNA viruses, nor are they limited to diagnosis of mystery illnesses or surveillance of disease-causing agents in mosquitoes. It is foreseeable that in the future this technology could be integrated into environmental sensors for biological warfare agents or other environmental contaminants, investigations of food or water-borne outbreaks, or a number of other applications.

There are many barriers to the use of routine metagenomic sequencing. Data handling and bioinformatic workflows need to be streamlined for non-expert users. For this approach to ever become part of the public health arsenal, our calculations suggest a need for generating tens of millions of high quality reads, ideally greater than 200 bases in length. While today this may not be cost-effective, given the very dramatic improvements in sequencing technology over the past 5 years [39], the day may come when sequencing wild caught mosquitoes to screen their viral load becomes a relatively routine procedure.

### Disclaimers

The views expressed in this article are those of the authors and do not necessarily reflect the official policy or position of the Department of the Navy, Department of Defense, United States Army, nor the U.S. Government. Some of the authors are military service members or employees of the U.S. Government. This work was prepared as part of their official duties. Title 17 U.S.C. §105 provides that ‘Copyright protection under this title is not available for any work of the United States Government.’ Title 17 U.S.C. §101 defines a U.S. Government work as a work prepared by a military service member or employee of the U.S. Government as part of that person's official duties.

## Supporting Information

Table S1 Top 100 transcripts detected from sequencing each of the 3 mosquito libraries are listed and are ranked by relative expression levels.
(0.03 MB XLS)

Table S2 Expression values and other characteristics, such as length and uniqueness, of transcripts detected from sequencing N2173 library (5 DENV-1 infected mosquitoes) are listed.
(1.79 MB XLS)

Table S3 Expression values and other characteristics, such as length and uniqueness, of transcripts detected from sequencing N2175 library (1 DENV-1 infected mosquito amongst 4 noninfected control mosquitoes) are listed.
(1.79 MB XLS)

Table S4 Expression values and other characteristics, such as length and uniqueness, of transcripts detected from sequencing N2187 library (5 noninfected control mosquitoes) are listed.
(2.10 MB XLS)

Table S5 Location of mosquito rRNA genes: results from BLAST of MQSRNAGN loci (Aedes albopictus rRNA genes) against *Ae. aegypti* published genome sequence.
(0.06 MB CSV)

Table S6 Mapping of N2173 reads: reads obtained from sequencing N2173 library (5 DENV-1 infected mosquitoes) were mapped to the *Ae. aegypti* published genome sequence (top) and cross-matched against rRNA gene-containing contigs (bottom).
(0.56 MB XLS)

Table S7 Mapping of N2175 reads: reads obtained from sequencing N2175 library (1 DENV-1 infected mosquito amongst 4 noninfected control mosquitoes) were mapped to the *Ae. aegypti* published genome sequence (top) and cross-matched against rRNA gene-containing contigs (bottom).
(0.70 MB XLS)

Table S8 Mapping of N2187 reads: reads obtained from sequencing N2187 library (5 noninfected control mosquitoes) were mapped to the *Ae. aegypti* published genome sequence (top) and cross-matched against rRNA gene-containing contigs (bottom).
(1.73 MB XLS)

Table S9 Calculation of pIP for each mosquito sample infected with DENV-1
(0.03 MB DOC)

Table S10 Metagenomic content of the mosquito samples was explored using an online automated metagenomic analysis server called MG-RAST. Specific taxa reported here are those to which a specific library had 50 or more hits.
(0.02 MB XLS)
