# Supplementary material for: Arbovirus Detection in Insect Vectors by Rapid, High-Throughput Pyrosequencing
Source: PLoS Negl Trop Dis. 2010 Nov 9;4(11):e878. doi: 10.1371/journal.pntd.0000878 (PMC2976685; doi:10.1371/journal.pntd.0000878)
Supplement: Table S9 — Calculation of pIP for each mosquito sample infected with DENV-1 (0.03 MB DOC) [file pntd.0000878.s009.doc]

| Sample | 1. Total reads | 2. DENV-1 reads | 3. Proportion  DENV-1 (column 2 divided by col 1) | 4. DENV-1 genomes per mosquito | 5. **p^IP^** (column 3 divided by column 4) | 6. DENV-1 Titer (MK-2 cells) |
| --- | --- | --- | --- | --- | --- | --- |
| N2173 | 289,436 | 227 | 7.8E-4 | 2.85E4 | **2.75E-8** | 1E4.9 |
| N2175 | 216,164 | 86 | 3.9E-4 | 1.05E4 | **3.79E-8** | 1E4.1 |
| N2473 | 390,971 | 243 | 6.2E-4 | 1.15E4 | **5.40E-8** | 1E5.2 |
| N2474 | 336,822 | 419 | 1.2E-3 | 1.15E4 | **1.08E-7** | 1E5.2 |
